# Silver Nanoparticles: Synthesis and Application for Nanomedicine

**DOI:** 10.3390/ijms20040865

**Published:** 2019-02-17

**Authors:** Sang Hun Lee, Bong-Hyun Jun

**Affiliations:** 1 Department of Bioengineering, University of California Berkeley, Berkeley, CA 94720, USA; shlee.ucb@gmail.com; 2 Department of Bioscience and Biotechnology, Konkuk University, 1 Hwayang-dong, Gwanjin-gu, Seoul 143-701, Korea

**Keywords:** silver nanomaterial, synthesis, characterization, mechanism, cytotoxicity, nanomedicine, diagnostics, optoelectronics

## Abstract

Over the past few decades, metal nanoparticles less than 100 nm in diameter have made a substantial impact across diverse biomedical applications, such as diagnostic and medical devices, for personalized healthcare practice. In particular, silver nanoparticles (AgNPs) have great potential in a broad range of applications as antimicrobial agents, biomedical device coatings, drug-delivery carriers, imaging probes, and diagnostic and optoelectronic platforms, since they have discrete physical and optical properties and biochemical functionality tailored by diverse size- and shape-controlled AgNPs. In this review, we aimed to present major routes of synthesis of AgNPs, including physical, chemical, and biological synthesis processes, along with discrete physiochemical characteristics of AgNPs. We also discuss the underlying intricate molecular mechanisms behind their plasmonic properties on mono/bimetallic structures, potential cellular/microbial cytotoxicity, and optoelectronic property. Lastly, we conclude this review with a summary of current applications of AgNPs in nanoscience and nanomedicine and discuss their future perspectives in these areas.

## 1. Introduction

Metal nanoparticles have been used in a wide-ranging application in various fields. Specifically, as shapes, sizes, and compositions of metallic nanomaterials are significantly linked to their physical, chemical, and optical properties, technologies based on nanoscale materials have been exploited in a variety of fields from chemistry to medicine [1,2,3]. Recently, silver nanoparticles (AgNPs) have been investigated extensively due to their superior physical, chemical, and biological characteristics, and their superiority stems mainly from the size, shape, composition, crystallinity, and structure of AgNPs compared to their bulk forms [4,5,6,7,8]. Efforts have been made to explore their attractive properties and utilize them in practical applications, such as anti-bacterial and anti-cancer therapeutics [9], diagnostics and optoelectronics [10,11,12], water disinfection [13], and other clinical/pharmaceutical applications [14]. Silver has fascinating material properties and is a low-cost and abundant natural resource, yet the use of silver-based nanomaterials has been limited due to their instability, such as the oxidation in an oxygen-containing fluid [15]. AgNPs, therefore, have an unrealized potential compared to relatively stable gold nanoparticles (AuNPs) [6]. Previous discoveries have shown that the physical, optical, and catalytic properties of AgNPs are strongly influenced by their size, distribution, morphological shape, and surface properties which can be modified by diverse synthetic methods, reducing agents and stabilizers [8,16]. The size of AgNPs can be adjusted according to a specific application—e.g., AgNPs prepared for drug delivery are mostly greater than 100 nm to accommodate for the quantity of drug to be delivered. With different surface properties, AgNPs can also be formed into various shapes, including rod, triangle, round, octahedral, polyhedral, etc [17]. Moreover, AgNPs are used in antimicrobial applications with proven antimicrobial characteristics of Ag^+^ ions. These exceptional properties of AgNPs have enabled their use in the fields of nanomedicine, pharmacy, biosensing, and biomedical engineering.

In this review, we present a comprehensive and contemporaneous view of the synthesis of AgNPs by various physio-chemical and biological methods, as well as the mechanism of action based on their unique properties. In addition, the review focuses on the characteristics of the optical and physio-chemical properties of AgNPs. Further, insights into understanding how various factors affect these distinct characteristics are discussed. Finally, promising applications of AgNPs in the biomedical field from nanomedicine to optoelectronics, including their anti-cancer or anti-bacterial activity, are presented.

## 2. Synthesis and Characterization of AgNPs

### 2.1. Synthesis of AgNPs via Top-Down and Bottom-Up Methods

**Figure 1:**
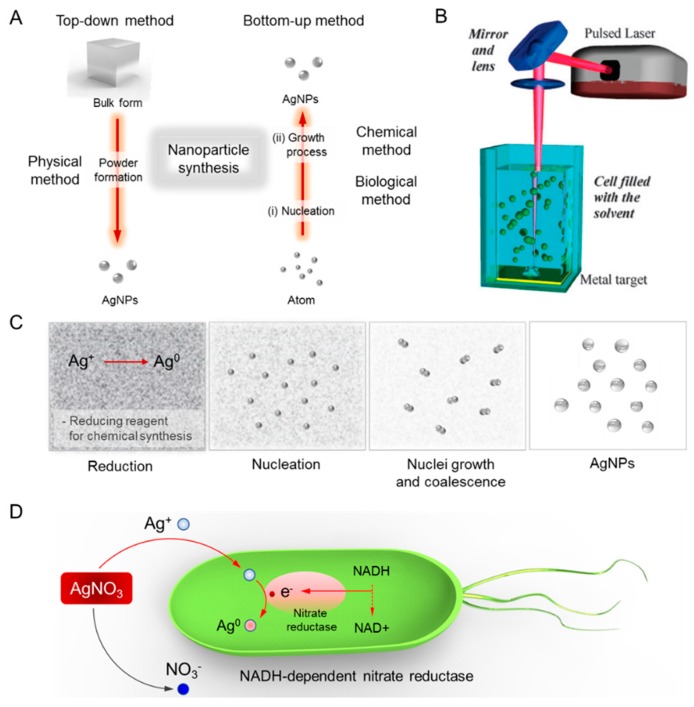
Diverse synthesis routes of silver nanoparticles (AgNPs). (**A**) Top-down and bottom-up methods. (**B**) Physical synthesis method. Reprinted with permission from [21]. Copyright 2009 Royal Chemical Society. (**C**) Chemical synthesis method. (**D**) Plausible synthesis mechanisms of green chemistry. The bioreduction is initiated by the electron transfer through nicotinamide adenine dinucleotide (NADH)-dependent reductase as an electron carrier to form NAD^+^. The resulting electrons are obtained by Ag^+^ ions which are reduced to elemental AgNPs.

As mentioned above, numerous types of silver nanostructures with distinctive properties have been used in various biomedical fields [18]. In particular, silver nanomaterials of varying sizes and shapes have been utilized in a broad range of applications and medical equipment, such as electronic devices, paints, coatings, soaps, detergents, bandages, etc [19]. Specific physical, optical, and chemical properties of silver nanomaterials are, therefore, crucial factors in optimizing their use in these applications. In this regard, the following details of the materials are important to consider in their synthesis: surface property, size distribution, apparent morphology, particle composition, dissolution rate (i.e., reactivity in solution and efficiency of ion release), and types of reducing and capping agents used. The synthesis methods of metal NPs are mainly divided into top-down and bottom-up approaches as shown in Figure 1A. The top-down approach disincorporates bulk materials to generate the required nanostructures, while the bottom-up method assembles single atoms and molecules into larger nanostructures to generate nano-sized materials [20]. Nowadays the synthetic approaches are categorized into physical, chemical, and biological green syntheses. The physical and chemical syntheses tend to be more labor-intensive and hazardous, compared to the biological synthesis of AgNPs which exhibits attractive properties, such as high yield, solubility, and stability [14]. The following sections discuss diverse synthesis methods in detail, from the synthesis of spherical AgNPs to shape-controlled Ag colloids, as well as how size-controlled AgNPs are synthesized. The sections also aim to introduce various routes of synthesis and their mechanisms, elucidating how shape- and size-controlled synthesis of AgNPs can be achieved through appropriate selection of energy source, precursor chemicals, reducing and capping agent, as well as concentration and molar ratio of chemicals.

### 2.2. Physical Method

The physical synthesis of AgNP includes the evaporation–condensation approach and the laser ablation technique [21,22]. Both approaches are able to synthesize large quantities of AgNPs with high purity without the use of chemicals that release toxic substances and jeopardize human health and environment. However, agglomeration is often a great challenge because capping agents are not used. In addition, both approaches consume greater power and require relatively longer duration of synthesis and complex equipment, all of which increase their operating cost.

The evaporation–condensation technique typically uses a gas phase route that utilizes a tube furnace to synthesize nanospheres at atmospheric pressure. Various nanospheres, using numerous materials, such as Au, Ag, and PbS, have been synthesized by this technique [23]. The center of the tube furnace contains a vessel carrying a base metal source which is evaporated into the carrier gas, allowing the final synthesis of NPs. The size, shape, and yield of the NPs can be controlled by changing the design of reaction facilities. Nevertheless, the synthesis of AgNPs by evaporation–condensation through the tube furnace has numerous drawbacks. The tube furnace occupies a large space, consumes high energy elevating the surrounding temperature of the metal source, and requiresa a longer duration to maintain its thermal stability. To overcome these disadvantages, Jung et al. demonstrated that a ceramic heater can be utilized efficiently in the synthesis of AgNPs with high concentration [24].

Another approach in physical synthesis is through laser ablation. The AgNPs can be synthesized by laser ablation of a bulk metal source placed in a liquid environment as shown in Figure 1B. After irradiating with a pulsed laser, the liquid environment only contains the AgNPs of the base metal source, cleared from other ions, compounds or reducing agents [25]. Various parameters, such as laser power, duration of irradiation, type of base metal source, and property of liquid media, influence the characteristics of the metal NPs formed. Unlike chemical synthesis, the synthesis of NPs by laser ablation is pure and uncontaminated, as this method uses mild surfactants in the solvent without involving any other chemical reagents [20].

### 2.3. Chemical/Photochemical Methods

Chemical synthesis methods have been commonly applied in the synthesis of metallic NPs as a colloidal dispersion in aqueous solution or organic solvent by reducing their metal salts. Various metallic salts are used to fabricate corresponding metal nanospheres, such as gold, silver, iron, zinc oxide, copper, palladium, platinum, etc. [26]. In addition, reducing and capping agents can easily be changed or modified to achieve desired characteristics of AgNPs in terms of size distribution, shape, and dispersion rate [27]. The AgNPs are chemically synthesized mainly through the Brust–Schiffrin synthesis (BSS) or the Turkevich method [20,28,29,30]. The strength and type of reducing agents and stabilizers should be taken into consideration in synthesizing metal NPs of a specific shape, size, and with various optical properties. More importantly, as stabilizing agents are typically used to avoid aggregation of these NPs, the following factors need to be considered for the safety and effectiveness of the method: choice of solvent medium; use of environment-friendly reducing agent; and selection of relatively non-toxic substances.

Nucleation and growth of NPs are governed by various reaction parameters, including reaction temperature, pH, concentration, type of precursor, reducing and stabilizing agents, and molar ratio of surfactant/stabilizer and precursor [31]. The chemical reduction of these metal salts can be accomplished by various chemical reductants, including glucose (C_6_H_12_O_6_), hydrazine (N_2_H_4_), hydrazine hydrate, ascorbate (C_6_H_7_NaO_6_), ethylene glycol (C_2_H_6_O_2_), N-dimethylformamide (DMF), hydrogen, dextrose, ascorbate, citrate (Turkevich method), and sodium borohydride (BSS method) [32,33]. Brust and co-workers have invented the most widely used synthesis method in producing thiol-stabilized AuNPs and AgNPs [30]. As shown in Figure 1C, silver ion (Ag^+^) is reduced in aqueous solution, receiving an electron from a reducing agent to switch from a positive valence into a zero-valent state (Ag^0^), followed by nucleation and growth. This leads to coarse agglomeration into oligomeric clusters to yield colloidal AgNPs. Previous studies using a strong reductant (i.e., borohydride) have demonstrated the synthesis of small monodispersed colloids, but it was found to be difficult to control the generation of larger-sized AgNPs. Utilizing a weaker reductant, such as citrate, resulted in a slower reduction rate, which was more conducive to controlling the shape and size distribution of NPs [34].

Stabilizing dispersive NPs during a course of AgNP synthesis is critical. The most common strategy is to use stabilizing agents that can be absorbed onto the surface of AgNPs, avoiding their agglomeration [35]. To stabilize and to avoid agglomeration and oxidation of NPs, capping agents/surfactants can be used, such as chitosan, oleylamine gluconic acid, cellulose or polymers, such as poly *N*-vinyl-2-pyrrolidone (PVP), polyethylene glycol (PEG), polymethacrylic acid (PMAA) and polymethylmethacrylate (PMMA) [27]. Stabilization via capping agents can be achieved either through electrostatic or steric repulsion. For instance, electrostatic stabilization is usually achieved through anionic species, such as citrate, halides, carboxylates or polyoxoanions that adsorb or interact with AgNPs to impart a negative charge on the surface of AgNPs. Therefore, the surface charge of AgNPs can be controlled by coating the particles with citrate ions to provide a strong negative charge. Compared to using citrate ions, using branched polyethyleneimine (PEI) creates an amine-functionalized surface with a highly positive charge. Other capping agents also provide additional functionality. Polyethylene glycol (PEG)-coated nanoparticles exhibit good stability in highly concentrated salt solutions, while lipoic acid-coated particles with carboxyl groups can be used for bioconjugation.

On the other hand, steric stabilization can be achieved by the interaction of NPs with bulky groups, such as organic polymers and alkylammonium cation that prevent aggregation through steric repulsion. For instance, Oliveira et al. described a Brust synthesis-modified procedure for dodecanethiol-capped AgNPs, wherein dodecanethiol could bind onto the surface of nanoparticles and exhibited high solubility without their aggregation in aqueous solution [36]. A phase transfer of a Au^3+^ complex can be carried out from aqueous to organic solution in a two-phase liquid–liquid system, then the complex can be reduced with sodium borohydride (NaBH_4_) along with dodecanethiol as a stabilization agent. The authors demonstrated that small alterations in parameters can lead to dramatic modifications in the structure, average size, and size distribution of the nanoparticles as well as their stability and self-assembly patterns [16].

Next, the surface of AgNPs conjugated with biomolecules, such as DNA probes, peptides or antibodies, can be used as a target for specific cells or cellular components. Attaching biomolecules to AgNPs can be achieved, for instance, by physisorption onto the surface of NPs or through covalent coupling by ethyl(dimethylaminopropyl) carbodiimide (EDC) to link free amines on antibodies to carboxyl groups. The photochemical synthesis method also offers a reasonable potential for the synthesis of shape- and size-controlled AgNPs although multiple synthesis steps may be required. Ag nanoprisms can be synthesized by irradiating Ag seed solution with a light at a selected wavelength. Commonly, the synthesis of bipyramids, nanodiscs, nanorods, and nano-decahedron involves a two-step process. Ag seeds prepared in the first step are subsequently grown in the second step by using an appropriate growth solution, by selecting a specific wavelength of light for irradiation, or by adjusting the duration of microwave irradiation. To synthesize distinctively shaped AgNPs, selective adsorption of surfactants/stabilizers to specific crystal facets needs to be controlled, since surfactants/stabilizers can guide growth along a specific crystal axis, generating varied shapes of AgNPs. The absorbance spectra of AgNPs have been reported to reflect changes in the shape of AgNPs. Such changes in UV–Vis–NIR spectra were illustrated during the photochemical synthesis of Ag nanoprisms grown by illuminating small silver NP seeds (λ_max_ of 397 nm) with low intensity LED [32]. As the seeds were converted to nanoprisms, the peak wavelength at 397 nm decreased over time, and new peaks appeared at 1330 nm and 890 nm, representing a localized surface plasmon resonance (LSPR) of the nanoprisms. For instance, the Mirkin group have investigated photo-induced conversion of spherical AgNPs to triangular prisms. Spontaneous oxidative dissolution of small Ag particles enabled the production of Ag^+^ ions that could subsequently be reduced on the surface of Ag particles by citrate under visible light irradiation [37].

### 2.4. Green Chemistry

Recently, the biogenic (green chemistry) metal NP synthesis method that employs biological entities, such as microorganisms and plant extracts, has been suggested as a valuable alternative to other synthesis routes as illustrated in Figure 1D [5,38,39]. It is known that microorganisms, such as bacteria and fungi, play a vital role in remediation of toxic materials by reducing metal ions [40,41]. Quite a few bacteria have shown the potential to synthesize AgNPs intracellularly, wherein intracellular components serve as both reducing and stabilizing agents [42]. The green synthesis of AgNPs with naturally occurring reducing agents could be a promising method to replace more complex physiochemical syntheses since the green synthesis is free from toxic chemicals and hazardous byproducts and instead involves natural capping agents for the stabilization of AgNPs [16].

A plausible mechanism of AgNP formation by the green synthesis was explored in the biological system of a fungus, *Verticillium species* [43,44]. The main hypothesis was that AgNPs are formed underneath the surface of the cell wall, not in the aqueous solution. Ag^+^ ions are trapped on the surface of the fugal cells due to the electrostatic interaction between Ag^+^ ions and negatively-charged carboxylate groups of the enzyme. Then, as intracellular reduction of Ag^+^ ions occurs in the cell wall, Ag nuclei are formed, which subsequently expand by further reduction of Ag^+^ ions. The result of transmission electron microscopy (TEM) analysis indicated that AgNPs were formed in cytoplasmic space due to the bioreduction of the Ag^+^ ions [45], yielding a particle size of 25 ± 12 nm in diameter. Interestingly, the fungal cells continued to proliferate after the biosynthesis of AgNPs. Bacteria commonly use nitrate as a major source of nitrogen, whereby nitrate is converted to nitrite by nitrate reductase, utilizing the reducing power of a reduced form of nicotinamide adenine dinucleotide (NADH). Bacterial metabolic processes of utilizing nitrate, namely reducing nitrate to nitrile and ammonium, could be exploited in bioreduction of Ag^+^ ions by an intracellular electron donor [46]. In fact, the utilization of nitrate reductase as a reducing agent is found to play a key role in the bioreduction of Ag^+^ ions [47]. For instance, Kumar and colleagues have demonstrated a rationale of an in vitro enzymatic strategy for the synthesis of AgNPs, based on α-NADPH-dependent nitrate reductase and phytochelatin [48]. Nitrate reductase purified from a fungus, *Fusarium oxysporum*, was used in vitro in the presence of a co-factor, α-NADPH. The process of AgNPs formation required the reduction of α-NADPH to α-NADP^+^. Hydroxyquinoline probably acted as an electron shuttle, transferring electrons generated during the reduction of nitrate to allow conversion of Ag^2+^ ions to Ag. As the Ag^+^ ions were reduced in the presence of nitrate reductase, a stable silver hydrosol (10–25 nm) was formed and subsequently stabilized by capping peptide. Similarly, AgNPs have been synthesized in various shapes using naturally occurring reducing agents (i.e., supernatants) in *Bacillus* species [49]. In *Bacillus licheniformis*, it was demonstrated that electrons released from NADH were able to drive the reduction of Ag^+^ ions to Ag^0^ and led to the formation of AgNPs. Li et al. also showed the synthesis of AgNPs by reductase enzymes secreted from a fungus, *Aspergillus terreus*, based on a similar NADH-mediated mechanism [50]. The synthesized AgNPs were polydispersed nanospheres ranging from 1 to 20 nm in diameter and exhibited antimicrobial potential to various pathogenic bacteria and fungi. In another example, *Pseudomonas stuzeri* isolated from a silver mine was used for the synthesis of AgNPs in aqueous AgNO_3_ [51]. The synthesized AgNPs exhibited a well-defined size and distinct morphology within the periplasmic space of the bacteria.

## 3. Characterization and Property of AgNPs

### 3.1. Plasmonic Properties

**Figure 2:**
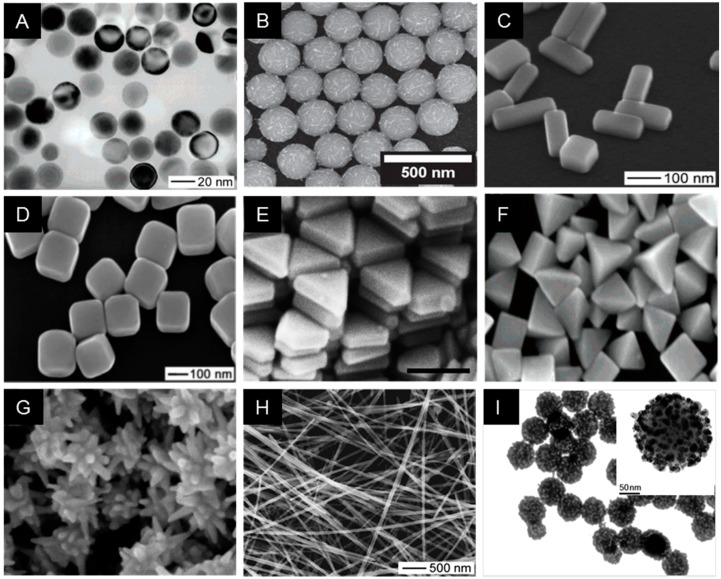
Representative images of electron microscopy of synthesized Ag nanostructures, demonstrating that diverse sizes and morphologies are made possible by controlling the reaction chemistry. (**A**) Silver nanosphere [58], (**B**) Silver necklaces [59], (**C**) Silver nanobars [60], (**D**) Silver nanocubes [7], (**E**) Silver nanoprism [61], (**F**) Silver bipyramids [62], (**G**) Silver nanostar [63], (**H**) Silver nanowire [58], (I) Silver nanoparticle embedded silica particle [64]. All figures were reprinted with permission from the publisher of each article.

**Figure 3:**
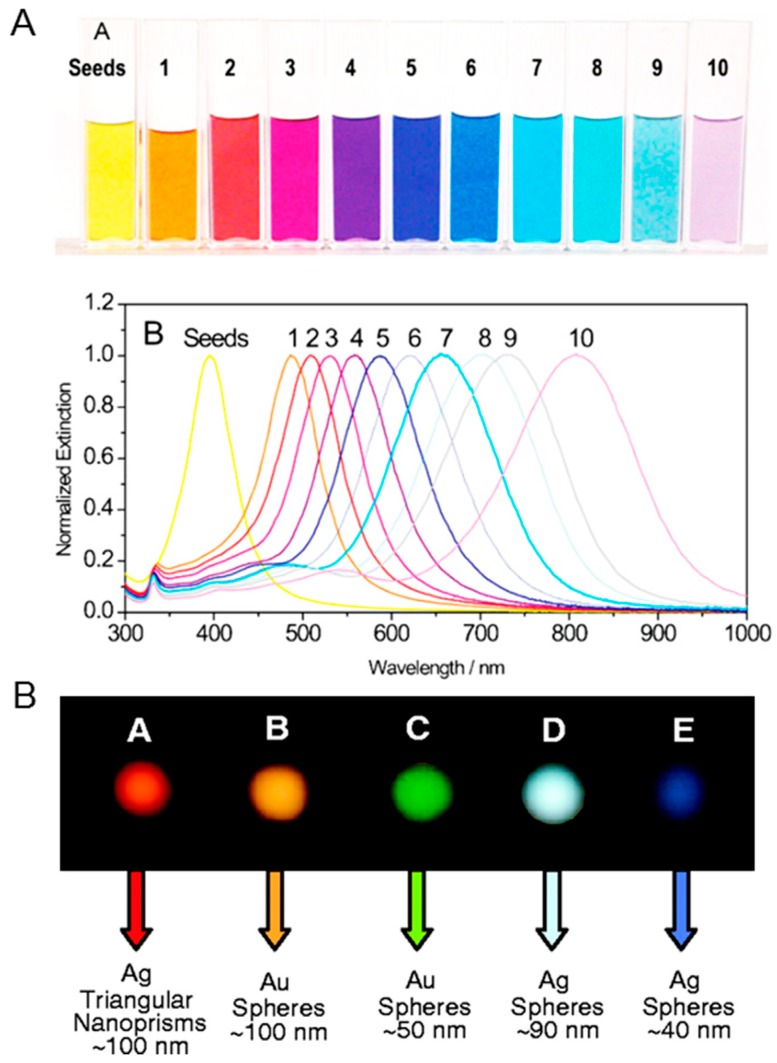
(**A**) Photograph of silver nanoprisms (top) and corresponding optical spectra changes of nanoprisms (bottom). Control on the edge-length of nanoprisms allows the plasmon resonance to be tuned across the visible and near-infrared portions of the spectrum. Reprinted with permission from [55]. Copyright 2008 Wiley-VCH. (**B**) Dark field microscopy images of (left to right) 100 nm diameter silver triangular nanoprism, 90 nm diameter silver nanosphere, and 40 nm diameter silver nanosphere, illustrating the ability to tune the scattering color of silver nanoparticle labels based on size and shape. Reprinted with permission from [56]. Copyright 2001 American Association for the Advancement of Science.

In many applications, surface chemistry, morphology, and optical properties associated with each NP variant require a careful selection to acquire the desired functionality of nanomaterials. In particular, corresponding reaction conditions during the synthesis of silver nanomaterials can be tuned to produce colloidal AgNPs with various morphologies, including monodisperse nanospheres, triangular nanoprisms, nanoplates, nanocubes, nanowires, and nanorods (Figure 2). Nowadays, since the most commonly used Ag and Au nanospheres are isotropic, they are widely utilized nanostructures for nanoantenna, capitalizing the LSPR phenomena caused by the collective oscillation of electrons in a specific vibrational mode at the conduction band near the particle surface in response to light. The optical properties can be varied by changing the composition, size, and shape of NPs which can affect the collective oscillation of free electrons in metallic NPs at their LSPR wavelengths when irradiated with resonant light over most visible and near-infrared regions [52,53]. Endowed with the tunable optical response, the NPs can be utilized as highly bright reporter molecules, efficient thermal absorbers, and nanoscale antenna, all through amplifying the strength of a local electromagnetic field to detect changes in the environment. The shape of silver nanoprisms has a specific peak wavelength that ranges from 400 to 850 nm as a surface plasmon resonance (SPR) band as shown in Figure 3A [54,55]. The SPR band or absorption spectra for nanoprisms can be measured by the UV–VIS spectroscopy, whereby the λ_max_ reflects an alteration in the size, shape, and the scattering color of AgNPs (Figure 3B) [56]. The optical properties of AgNPs have been of particular interest due to the strong coupling of AgNPs to specific wavelengths of incident light. Ag nanospheres are known to have rather short LSPR wavelengths in the violet and blue regions of the visible spectrum.

AgNPs can be utilized in bio-sensing by single nanoparticle spectroscopy, such as dark-field microscopy. Alivisatos and his co-worker described ‘plasmon rulers’ to monitor distances between two distinct nanoparticles [57]. The distance can be determined by plasmonic coupling of two nanospheres modified at two ends of a single-stranded DNA (ssDNA) probe with biotin on one end and streptavidin on the other end. The authors demonstrated the plasmonic coupling between single pairs of silver and gold nanoparticles to measure the DNA length and tracked the hybridization kinetics over 3000 s. The plasmonic coupling between two distinct nanoparticles led to more pronounced spectral changes based on the dimerization of single nanoparticles. For example, DNA hybridization was responsible for the observed blue-shift in the spectra via an increase in steric repulsion or for the observed drastic red-shift via aggregation, such as DNA wrapping around DNA-binding dendrimers. In addition, the distance between the AuNPs was adjusted by controlling the length of ssDNA and by changing the ionic strength of the buffer. The maximum plasmon resonance (LSPR) shifted to the red region at high salt concentrations (0.1 M NaCl), indicating a decreased distance between the two AuNPs due to the reduced electrostatic repulsion of the particles at high ionic strength environments. Conversely, low salt concentrations (0.005 M NaCl) increased electrostatic repulsions and led to the blue shift of the maximum LSPR. Along with these results, the hybridization of complementary DNA also resulted in a significant blue shift, which is expected considering that the structural property of double-stranded DNA (dsDNA) shows greater stiffness than ssDNA, and hence allowing it to repulse two AuNPs.

Several reports have demonstrated that AgNPs absorb electromagnetic radiation in the visible range from 380 to 450 nm, which is known as the excitation of LSPR. The optical properties of AgNPs of different sizes by gallic acid using biological synthesis methods were characterized by Park and colleagues [65]. The authors demonstrated that spherical AgNPs of 7 nm have SPR at 410 nm, while those of 29 nm have 425 nm. In addition, 89 nm-sized AgNPs exhibited a wider band with a maximum resonance at 490 nm. It was noticed that the width of the SPR band was related to the size distributions of NPs. For instance, Lee et al. investigated the dependence in the sensitivity of SPR responses (frequency and bandwidth) that enabled NPs to recognize the changes in their surrounding environment. They also demonstrated how the optical scattering of Au or Ag nanorods with diverse sizes and shapes can affect total extinction [66]. Greater enhancement in the magnitude and sharpness of the plasmon resonance band was observed in nanorods with higher Ag concentration, which could contribute to superior sensing resolution even with a similar plasmon response. As such, Ag nanorods have an additional advantage as better scatterers when compared to Au nanorods.

### 3.2. Chemical Cytotoxicity

One of the current issues in AgNP-based nanomedicine involves nanotoxicity and environmental impact of AgNPs on a nanometer scale. To predict the potential cytotoxic effect of AgNPs, it is necessary to investigate chemical transformation that occurs with AgNPs travelling through the intracellular environment [15]. The use of AgNPs based on their chemical cytotoxic property has received much attention as potent anticancer or antibacterial agents. Despite various hypotheses available, the mechanisms of the antibacterial properties of AgNPs so far have not been established clearly. Based current literature, the proposed cytotoxic mechanisms can be summarized as follows: (i) adhesion of AgNPs onto the membrane surface of microbial cells, modifying the lipid bilayer or increasing the membrane permeability; (ii) intracellular penetration of AgNPs; (iii) AgNP-induced cellular toxicity triggered by the generation of reactive oxygen species (ROS) and free radicals, damaging the intracellular micro-organelles (i.e., mitochondria, ribosomes, and vacuoles) and biomolecules including DNA, protein, and lipids; and (iv) modulation of intracellular signal transduction pathways towards apoptosis. Critical parameters, such as ion release, surface area, surface charge, concentration and colloidal state, can all influence the cytotoxic properties of AgNPs.

**Figure 4:**
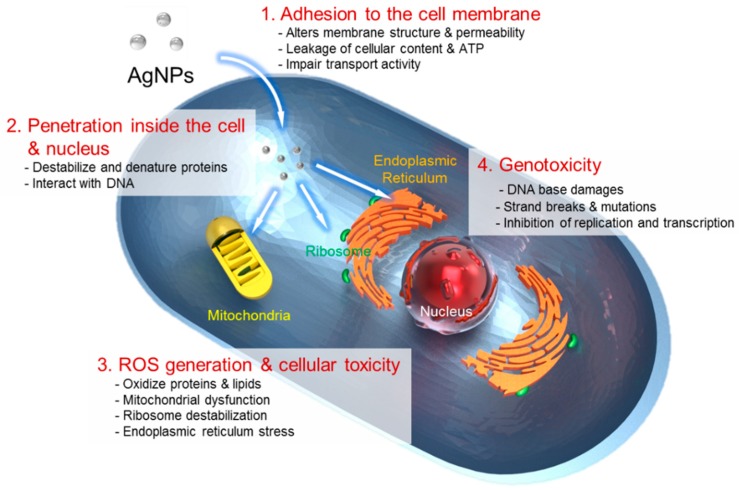
The four main routes of cytotoxic mechanism of AgNPs. 1, AgNPs adhere to the surface of a cell, damaging its membrane and altering the transport activity; 2, AgNPs and Ag ions penetrate inside the cell and interact with numerous cellular organelles and biomolecules, which can affect corresponding cellular function; 3, AgNPs and Ag ions participate in the generation of reactive oxygen species (ROS) inside the cell leading to a cell damage and; 4, AgNPs and Ag ions induce the genotoxicity.

The main mechanism of AgNPs regarding their antimicrobial activity can be simplified to their high surface area in releasing silver ions. AgNPs in an aqueous environment are oxidized in the presence of oxygen and protons, releasing Ag^+^ ions as the particle surface dissolves. The release rate of the Ag^+^ ions depends on a number of factors including the size and shape of NPs, capping agent, and colloidal state. For example, it is well known that antibacterial activity is enhanced with the release of Ag^+^ ions from AgNPs onto the bacterial cells [11]. In particular, smaller or anisotropic AgNPs with a larger surface area showed more toxicity and exhibited a faster ion release rate due to high surface energy originating from highly curved or strained shapes of NPs [67]. The small-sized AgNPs also exhibited a superior release rate of silver ion particularly into the Gram-negative bacteria. The shape and higher temperature of AgNPs equally caused a greater degree of toxicity and accelerated the rate of ion release by more effective dissolution [68,69]. Furthermore, the cytotoxic effect of AgNPs arises in a similar concentration range for both bacteria and human cells [70]. Therefore, a higher Ag ion concentration, a faster release rate of the Ag ions, and a larger surface area of AgNP should be considered for the enhanced antimicrobial treatment in clinical medicine [71]. Moreover, the presence of chlorine, thiols, sulfur, and oxygen was shown to strongly impact the rate of silver ion release [72]. Silver ions can interact with thiol groups in critical bacterial enzymes and proteins, and subsequently damage cellular respiration, resulting in cell death. The generation of ROS and free-radicals is another mechanism of AgNPs causing a cell-death process as illustrated in Figure 4. The potent cytotoxic activity of AgNPs, such as antibacterial, antifungal, and antiviral action, is mainly due to their ability to produce ROS and free radical species, such as superoxide anion (O_2_^−^), hydrogen peroxide (H_2_O_2_), hydroxyl radical (OH), hypochlorous acid (HOCI), and singlet oxygen [73]. When in contact with bacteria, the free radicals have the ability to generate pores on the cell wall, which can ultimately lead to cell death [10]. AgNPs can also anchor to the surface of the bacterial cell wall and penetrate it to cause structural changes to the membrane or increase its permeability, all of which trigger cells to die.

Interestingly, the strength of the antibacterial property of AgNPs is correlated with different types of bacterial species, such as Gram-positive and -negative bacteria. This is because these species differ in the architecture, thickness, and composition of their cell wall [74]. It is known that *Escherichia coli* (*E. coli*) which is Gram-negative bacteria is more susceptible to Ag^+^ ions than Gram-positive *Staphylococcus aureus* (*S. aureus*). The reason for different susceptibility lies on the peptidoglycan which is a key component of the bacterial cell membrane. The cell wall in Gram-positive bacteria is composed of a negatively-charged peptidoglycan layer with approximately 30 nm in thickness, whereas Gram-negative bacteria have a peptidoglycan layer of only 3 to 4 nm [31,75]. These structural differences, including the thickness and composition of the cell wall, explain why Gram-positive *S aureus* is less sensitive to AgNPs, and Gram-negative *E.coli* displays substantial inhibition even at a low concentration of AgNPs. Loo et al. investigated silver and curcumin NPs against both Gram-positive and Gram-negative bacteria, and the NPs in 100 µg/mL concentration were able to distort matured bacterial biofilms. The sustained anti-bacterial effects of this formulation can be utilized in antimicrobial treatment [76]. Petr Pařil et al. investigated the anti-fungal effects of AgNPs and copper nanospheres against wood-rotting fungi [77]. The AgNP treatment required a very low mass of NPs and exhibited high efficiency against *Tinea versicolor* (*T. versicolor*) fungi in comparison to *Poria placenta (P. placenta)* fungi, showing differing anti-fungal effects of AgNPs against white and brown-rot fungi, respectively. The details of antimicrobial properties are beyond the scope of this review and are reviewed elsewhere [9,14,72,78].

### 3.3. Alloy with Other Metals

Alloy NPs exhibit specific properties that are different from their individual NPs. They can be created directly by combining different metallic nanocrystals (NCs) in specific numbers and arrangements [79]. As strong electronic coupling exists between two metals, the bimetallic nanocrystals show more enhanced catalytic, electronic and optical properties compared to monometallic nanocrystals [80]. The properties of alloy NPs are defined by their internal configuration (i.e., arrangement of constituent atoms) and external structures, such as shapes and sizes. The LSPR wavelength of nanocrystals formed from Ag–Au alloys can be tuned by varying the Au:Ag ratio and, therefore, can increase gradually with an increase in the percentage of Au in the alloy nanocrystal. However, metallic atoms are easily bonded in a non-specific manner, lacking the directionality of covalent bonds and equivalence of molecules. The synthesis methods for alloy NPs can be divided into two categories: (i) successive/sequential reduction method and (ii) co-reduction/simultaneous reduction method with metal precursors [81].

**Figure 5:**
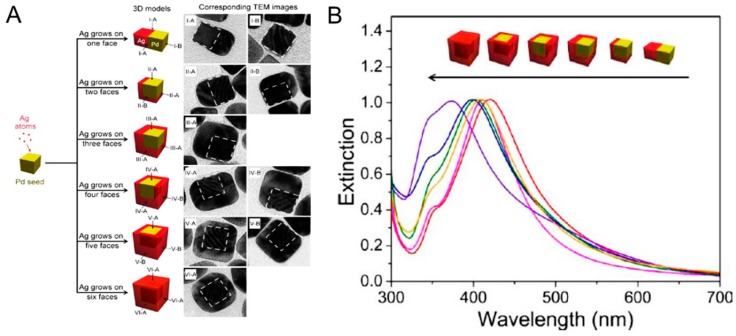
Controlled overgrowth of Ag for bimetallic nanocrystals. (**A**) Schematic illustration showing the site-selective growth of Ag on each cubic seed and corresponding transmission electron microscopy (TEM) images. Well-controlled bimetallic nanocrystals were fabricated along the directed size and number of facets on a cubic Pd seed. The white dashed lines in the TEM indicate the position of the cubic Pd seed. (**B**) Extinction spectra of the Pd–Ag bimetallic nanocrystals with Ag growing on different numbers of faces of the cubic Pd seed. The LSPR peak blue-shifted with the increase in the number of faces involved in the Ag growth. Reprinted with permission from [80]. Copyright 2012 American Chemical Society.

Sequential reduction without protective agents is driven thermodynamically and causes the formation of core–shell NPs or other types of hetero-nanostructures. The sequential reduction method involves subsequent seed-mediated growth of NPs with metal precursors and reducing agents over time. The bimetallic colloids with different metals, such as Ag and Au, can be synthesized in several different ways, resulting in Ag-coated Au or Au-coated colloidal particles. The synthesis can be done simply by reducing one metal salt on already-formed counterpart metal NPs—e.g., to synthesize Ag-coated Au colloids, chemically reduce silver salt on the AuNPs [82]. This seed-mediated synthesis method for core–shell and intermetallic structures is widely used in well-defined bimetallic NPs, due to its capability to regulate the size, shape and composition of the final compound [83,84,85]. Reducing agents also play a vital role in controlling the size distribution. The co-reduction method with different metal precursors to zero-valent atoms has made bimetallic colloids readily accessible [86]. The key advantages lie in the simplicity and versatility of the technique. Using this method, several types of Ag and Au bimetallic core–shell colloids with various shapes have been produced [87]. Bimetallic colloids with gradient metal distribution or with a layered structure are one of the most interesting and promising methods in catalytic applications. In the co-reduction method, however, composition uniformity is a major drawback due to the high prevalence of sequential reduction. For example, the Xia group at Georgia Institute of Technology described nucleation and site-selective growth of Ag on cubic Pd nanocrystal seeds as shown in Figure 5 [80]. Ag atoms were directed to nucleate in a specific-site and then grown on a specific number of faces on a cubic Pd nanocrystal seed by controlling reaction kinetics. This approach allowed the fabrication of bimetallic nanocrystals with well-controlled spatial distributions and tunable LSPR properties. Another example is from Lee and colleagues who demonstrated programmable synthesis of hybrid liposome-metal NPs which allows self-crystallization of metal NPs in liposome [88]. They have synthesized seven types of liposome/monometallic and liposome/bimetallic hybrids with Ag, Au, Pd, Pt, Ag–Au, Au–Pt, and Au–Pd, which were tunable in size and composition. The resulting NPs showed controllable SPR bands in visible and near-infrared spectra as well as better colloidal stability caused by an outer liposome structure. This improved physicochemical property allowed the liposome/NP hybrids to be applied to the intracellular imaging of living cells via SERS. On the other hand, the enhanced catalytic performance of bimetallic Au–Ag (core–shell) colloids on luminol-K_3_-Fe(CN)_6_ chemiluminescence (CL) has been described by Zhang and colleagues [89]. Bimetallic Au–Ag NPs were synthesized by a sequential two-step reduction technique, and subsequently prostate-specific antigen (PSA) specific antibody was conjugated on the Au–Ag NPs. Since the prepared Ag–Au NPs with synergistic catalytic activity significantly enhanced the CL reaction, the PSA antibody-Ag–Au NP immune complex was able to successfully applied to the detection of PSA in human serum sample down to 0.047 pg/mL (S/N = 3).

## 4. Applications of AgNPs

### 4.1. AgNP-Based Nanomedicine

#### 4.1.1. Plasmonic Nanoantennas

Recently, AgNP has been widely utilized in various subfields of nanomedicine including nanoelectronics, diagnostics, molecular imaging, and biomedicine. These interesting applications are based on utilizing an enhanced electromagnetic field on and near the surface of AgNPs. At the plasmon resonant wavelength, AgNPs act as nanoscale antennas, increasing the intensity of a local electromagnetic field. One spectroscopic technique that benefits from the enhanced electromagnetic field is the Raman spectroscopy, where molecules can be identified by their unique vibrational modes. However, intrinsic Raman scattering of photons from molecules is weak and requires a longer measurement duration to obtain a Raman spectrum. Therefore, surface-enhanced Raman scattering (SERS) from molecules near the surface of plasmonic nanoantenna offers great amplification of Raman signals. Typically, SERS detection involves adsorption of molecules on Ag or Au nanoparticle aggregates or solid substrates with plasmonic nanostructures [90,91]. Strong field enhancement is generated in the nanogaps or interstices known as hot spots within interacting plasmonic nanostructures [92]. The SERS effect can be used to detect critical proteins and biomolecules, such as early cancer biomarkers or drug levels in blood and other body fluids. Up until recently, the SERS effect with hot spots has been the main focus in numerous experimental and theoretical studies, which can enhance the Raman scattering to the factor of 10^8^ to 10^12^, allowing the detection of even a single molecule [93].

**Figure 6:**
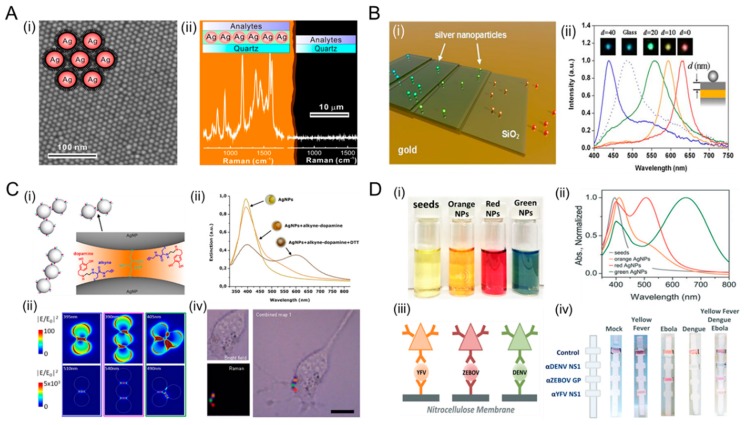
Plasmonic AgNPs for plasmonic nanoantennas and diagnostics. (**A**) Single-layer AgNP surface-enhanced Raman scattering (SERS) film for a large-scale hot spot. (**i**) Scanning electron microscopy (SEM) image of a superlattice of 6 nm. AgNPs were used as a homogeneous single-molecule SERS substrate. Illustration shows an interparticle gap for hot spots, which is regulated by the length of a thiolate chain. (**ii**) Two Raman spectra of single-layered SERS film (left) and quartz surface (right). The enhancement factor was estimated to be larger than 1.2 × 10^7^. Reprinted with permission from [94]. Copyright 2015 American Chemical Society. (**B**) Metal-film induced plasmon resonance tuning of AgNPs. (**i**) Schematic illustration of optical scattering spectra of AgNPs on different substrates. (**ii**) Single AgNP spectra of AgNPs on a silica spacer layer of varying thickness *d* (nm) on a glass substrate with a 50 nm gold film. The inset is a dark-field image of AgNPs with the corresponding color. The dotted lines represent single particle spectra of AgNPs on a plain glass substrate. Reprinted with permission from [103]. Copyright 2010 American Chemical Society. (**C**) SERS-based intracellular imaging using alkyne-AgNPs nanoprobes. (**i**) The structure of colloidal alkyne-AgNP clusters with nano-sized interparticle gaps. (**ii**) Extinction spectra of the alkyne-AgNPs nanoprobe. The resonance peaks at 400 nm shifted around 520 nm after metal functionalization. (**iii**) Computational simulation of the far- and near-field optical responses. Intensity distributions of the single particle mode (upper-panels) and the dimer mode (bottom-panels) (**iv**) Intracellular Raman imaging of a AgNP nanoprobe within the cytoplasmic space of fibroblast. Distinguishable hot spots were highlighted by color-dots related to Raman intensity of the akyne 2045 cm^−1^ band. Reprinted with permission from [104]. Copyright 2018 Nature Publishing Group. (**D**) Multiplexed detection with a tunable wavelength of AgNPs. (**i**) Different colors of AgNPs during a stepwise growth. (**ii**) Corresponding absorption spectra with varying sizes of AgNPs, such as 30, 41, and 47 nm. (**iii**) Individual testing of Yellow Fever virus (YFV) NS1 protein, Zaire Ebola virus (ZEBOV) glycoprotein (GP), and Dengue virus (DENV) NS protein using AgNPs. Orange, red, and green AgNPs were conjugated with monoclonal antibodies specific to YFV NS1, ZEBOV GP, and DENV NS, respectively. (**iv**) Multiplexed detection using different AgNPs-based lateral flow assays. Reprinted with permission from [105]. Copyright 2015 Royal Society of Chemistry.

Numerous approaches have been made to utilize the plasmonic property of AgNPs. For instance, techniques to control the distance and spacing of hot spots are essential in quantitative SERS covering large areas as shown in Figure 6A,B [94]. Strong enhancement of single hot spots may lead to a false representation of a sample when the signal is mainly determined by a few detection sites. Nanoparticle superlattices have demonstrated a potential to counterbalance the homogeneous distribution in sensing hot-spot bands and to enhance the detection performance of the sensor. Sun and colleagues described a SERS substrate via graphene–AgNPs heterojunction which improved Raman signals [95]. With increasing the density of AgNPs, Raman scattering of graphene-veiled AgNPs heterojunction substrate was significantly enhanced by approximately 67 folds compared to R6G analyte. The cooperative synergy generated by the coupling of graphene and deposited AgNPs can be utilized to create a strong electromagnetic hot spot for an optical sensing platform. Another example is a star-shaped Au/AgNPs SERS substrate on Ge (5 nm)/Ag (25 nm)/GE (75 nm)/glass (germanium–silver multilayers) which was employed via near-infrared (NIR) SERS operation by Lai and colleagues [96]. The hybrid SERS substrate was operated at a 1064 nm excitation and exhibited 30% higher Raman intensity.

AgNPs can be utilized as highly sensitive NP probes for targeting and imaging of small molecules, DNA, proteins, cells tissue, and even tumor in vivo (Figure 6C) [97,98]. AgNPs with stronger and sharper plasmon resonance have been widely used in imaging systems, particularly for cellular imaging with contrast agents functionalized to AgNPs via surface modification. For example, a AgNP-embedded nanoshell structure can be used in cancer imaging and photothermal therapy to explore the location of cancer cells by absorbing light and destroy them via photothermal effect [99]. Kang et al. described NIR-sensitive SERS nanoprobes for an in vivo multiplex molecular imaging to detect aromatic compounds [100]. The NIR SERS probes with plasmonic Au/Ag hollow-shell were assembled onto silica nanospheres which exhibited a red-shift of plasmonic extinction band in the NIR optical window region (700–900 nm). The signals from the NIR-SERS nanoprobe for a single particle detection exhibited a detectable signal from animal tissues that were 8 mm deep [101]. Jun et al. showed Ag-embedded SERS nanoprobes, called M-SERS dot, which have a Raman signature for imaging of target cancer cells as well as strong magnetic properties for identifying desirable cells [102]. AgNP-embedded magnetic nanoparticles (MNPs), which consisted of a magnetic core (18 nm) and a silica shell (16 nm thick) decorated with AgNPs on the surface, were prepared. The M-SERS dots exhibited strong SERS signals originating from diverse encoding materials, such as AgNPs and Raman-labels. The Ag-embedded M-SERS dots with highly sensitive SERS signals enabled targeting, isolation, and imaging of cancer cells. To investigate their specific targeting and sorting abilities, M-SERS conjugated with targeting antibodies were added into multiple cell population, and subsequently, the targeted cancer cells could easily be isolated by an external magnetic field. Hahm et al. described a multilayered core–shell nanoprobe with Ag-embedded silica nanostructure for a SERS-based chemical sensor [64]. The multi-layered nanoprobe consisted of a silica core coated with Raman label, silica shell, and AgNPs. The embedded inner AgNP and Raman label compound in the nanoprobe served as an internal standard for calibrating SERS signals, while the outer AgNPs were utilized as a sensing site for analyte detection. These chemical sensors based on the ratiometric analysis (*I_A_*_nalyte_/*I*_Internal standard_) could be applied to various SERS probes for quantitative detection of a wide variety of targets.

#### 4.1.2. Diagnostics with Tunable Wavelength

AgNPs can absorb and scatter light with extraordinary efficiency. A large scattering cross-section of the nanospheres allows for an individual AgNP to be imaged under a dark-field microscopy or hyperspectral imaging systems. As mentioned above, AgNPs have been intensively utilized in several applications, including diagnosis and bioimaging of cancer cells [106]. Furthermore, AgNPs have been utilized for the detection of p53 in carcinoma cells [107]. Zhang et al. demonstrated that nanostructures comprising silver cores and a dense layer of Y_2_O_3_:Er separated by a silica shell is an excellent system model to investigate the interaction between upconversion materials and metals on a nanoscale. Nanoparticles are also potentially promising as fluorescent labels for (single particle) imaging experiments or bioassays, which require low background or tissue penetrating wavelengths [108]. Optical properties of AgNPs can be utilized for multiplexed point-of-care (POC) diagnostics using their size-tunable absorption spectra. As shown in Figure 6D, Yen et al. described a multicolored AgNPs-based multiplexed lateral flow assay (LFA) for multiple pathogen detections [105]. Multiplexed rapid LFA diagnostics has the ability to discriminate among multiple pathogens, thereby facilitating effective investigations for diagnosis. Specifically, triangular plate-shaped AgNPs with varying sizes, such as 30 nm, 41 nm. and 47 nm, have narrow absorbance that are tunable through the visible spectrum, resulting in an easily distinguishable color. The multicolored AgNPs were conjugated with antibodies to recognize dengue virus (DENV) NS protein, Yellow Fever virus (YFV) NS1 protein, and Zaire Ebola virus (ZEBOV) glycoprotein (GP). The limit of detection (LOD) for the biomarkers of each virus was 150 ng/mL in a single channel. Another example is a colorimetric lead detection using AgNPs described by Balakumar and co-workers [109]. The synthesized AgNPs exhibited high sensitivity for the detection of as low as 5.2 nM of Pb^2+^ in the range of 50 to 800 nM and also showed selective recognition even in the presence of interfering metal ions. This approach can be used for a rapid and cost-effective detection of saturnism (lead poisoning) in a water sample.

#### 4.1.3. Surface-Enhanced Fluorescence

**Figure 7:**
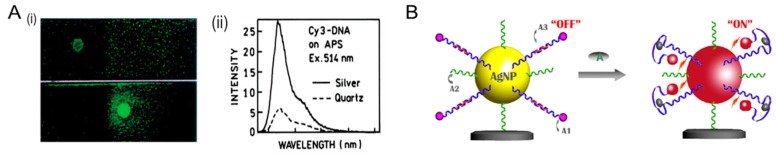
Surface-enhanced fluorescence. (**A**) Metal-enhanced fluorescence on a Ag film. (i) The photograph shows fluorescence spots on quartz (top) and silver (bottom) taken through 530 nm long pass filter for Cy3-DNA. (ii) Emission spectra of Cy3-DNA on APS-treated slides, with (solid line) and without silver island films (dotted line). Reprinted with permission from [113]. Copyright 2003 Future Science Group. (**B**) Schematic illustration of an aptamer-based AgNP nanosensor, showing the ‘off’ state via fluorophore quenching within short distances (left) and ‘on’ state via turn-on fluorescence signal (right) based on the spacing distance between the Cyanine 3 and the AgNP surface in the detection of adenosine. Reprinted with permission from [114]. Copyright 2012 Elsevier.

Surfaces of metallic nanoparticles can alter the free-space condition of fluorescence with spectral properties which can result in dramatic spectral changes as shown in Figure 7A [110]. This interactions between metal surface and fluorophore have been termed variously as surface-enhanced fluorescence (SEF), metal-enhanced fluorescence (MEF) or radiative decay engineering [111]. The metallic surfaces exhibit the following features as illustrated in Figure 7B: fluorophore quenching within short distances (0–5 nm); spatial disparity of incident light (0–15 nm); and changes in the radiative decay rates (1–20 nm). The enhanced field effect can be leveraged to build an interspace with a shorter distance between a fluorophore and the surface of a metal nanostructure composed of Ag or Au, increasing fluorophore emission rate [112]. The enhanced fluorescence can be attributed to two main factors, which are (i) an enhanced excitation rate by large absorption and scattering cross-section of the plasmonic nanoparticles to incoming light and (ii) a decrease in fluorescence lifetime of the fluorophore that allows an excited state to return to the ground state at a higher frequency. In particular, SEF parameters of the dye/nanoparticle coupled system are related to the distribution of near-field intensity and their distance-dependent decay function. Such effects depend strongly on the overlap of optical properties of the fluorophore and nanosphere and on the physical location of the fluorophore around the particles.

### 4.2. Biomedical Application of AgNPs

Owing to their intrinsic cytotoxicity, AgNPs have been broadly used as antibacterial and anticancer agents and for biomedical application in the healthcare industry. The degree of toxicity against cells is determined by the surface charges of AgNPs [115]. A positive surface charge of AgNPs renders them more suitable to stay for a longer duration on the tissue surface or luminal side of the blood vessel, which is a major route for administrating anticancer agents [116]. The intrinsic cytotoxic property of AgNPs has been applied against various types of cancer cells, such as hepatocellular carcinoma [117], lung [118] and breast cancer [119,120], and cervical carcinoma [121]. Small sized AgNPs were more efficient in ROS production [122]. Apart from these cellular mechanisms, AgNPs have also shown anti-angiogenic and anti-proliferative properties [123,124]. The anti-proliferative property mediated by AgNPs in cancer cells is due to their ability to damage DNA, break chromosome, produce genomic instability, and disrupt calcium (Ca^2+^) homeostasis which induces apoptosis and causes cytoskeletal instability. The cytoskeletal injury blocks the cell cycle and division, promoting anti-proliferative activity of cancer cells [89].

For instance, in regard to intracellular transport, Lee et al. characterized the transport of a single AgNP into an in vivo Zebrafish embryo model system and their effects on early embryonic development on a single-nanoparticle resolution in real-time. It was found that a single Ag nanoparticle (5–46 nm) was transported into and out of embryos through chorionic pore channels (CPCs) and exhibited Brownian diffusion (not an active transport). The diffusion coefficient inside the chorionic space (3 × 10^−9^ cm^2^/s) was ~26 times lower than that in egg water (7.7 × 10^−8^ cm^2^/s). Thapa et al. embedded graphene oxide in AgNPs (GO-AgNP) using glucose as a reducing agent. By covalent conjugation of methotrexate (MTX) to Go-AgNP via an amide bond, targeting of folate receptors expressing cancer cells was achieved, and, thus, showing that the combination of anticancer drug and AgNPs could be used synergistically for treatment of cancer [125]. As another example, Azizi and colleagues developed a novel nanocomposite with the aim of developing AgNPs as a new anticancer agent that specifically target tumor cells. Albumin coated AgNPs were synthesized, and their anti-cancerous effects were evaluated against MDA-MB 231, a human breast cancer cell. The cancer cell showed morphological changes, and its DNA agarose gel pattern on gel electrophoresis revealed a cell death process through apoptosis. It was found that AgNPs with a size of 90 nm and with a negative charge of a zeta-potential of about −20 mV could be specifically taken up by tumor cells. The LD_50_ of AgNPs against MDA-MB 231 (5 µM) suggests the AgNPs to be a good candidate as a chemotherapeutic drug [126]. In therapeutics outside oncology, Ayaz and colleagues have described AgNPs conjugated with anti-seizure drugs (as a drug carrier) against brain-eating amoebae (*Naegleria flowleri*) to treat central nervous system (CNS) infection [127]. Anti-seizure drugs which are known to cross the blood–brain barrier (BBB) were attached to the surface AgNPs as capping agents. AgNPs conjugated with drugs, such as diazepam, phenobarbitone, and phyenytoin, exhibited overall anti-amoebic activities against both trophozoite and cyst stages. Moreover, significant enhancement of fungicidal activities was shown against both trophozoite and cyst amoebic stages compared to those of the drugs alone. The researchers suggested that a feasible mechanism of AgNPs-based drugs which can penetrate BBB might lie in their ability to bind to the receptors and ion channels on the cell membrane of amoebae.

The cytotoxic effect of AgNPs has been used extensively in food and healthcare industries, such as food storage, textile, medical device coating, and environmental sensing [128]. Specific toxicity to bacteria has led to the integration of silver in a wide variety of products including wound dressings, packaging materials, and anti-fouling surface coatings. Another interesting approach is AgNP-coated bandages as they can kill harmful microbes and allow better healing at the injured tissue. In addition, silver ions as an antimicrobial agent have been used as composites in dental resin and in coatings of medical instruments [129]. AgNPs have also been utilized in food packaging so that foods can last for longer without contamination.

### 4.3. Optoelectronics

Diverse silver nanomaterials have been studied as components of nanocomposite due to their high dielectric constants in numerous systems. For example, silver nanowires can be used as conductive coatings in flexible electronics and transparent semiconductors [130]. Similarly, AgNPs have the potential to be applied in silver paste for efficient contact at electronic interfaces because of their high conductivity [10]. In particular, AgNPs can be used as antennas enhancing plasmonic activity for sensing of a specific molecule or in imaging applications. AgNPs can, therefore, be utilized as a sensing material for environmental monitoring [131]. Prosposito et al. reported that negatively-charged AgNPs with -34 mV in zeta-potential exhibited a good response to heavy metals, such as nickel (II) [132]. They synthesized AgNPs with an average diameter of 2.5 nm in water phase using silver nitrate as a precursor, hydrophilic thiol (3-MPS, 3-mercaptopropane sulfonate) as a capping agent, and sodium borohydride as a reducing agent. The SPR spectral changes in the presence of metal ions, such as Ni^2+^, Cr^3+^, Nd^3+^, Cu^2+^, and Ca^2+^, were observed. AgNP/3-MPS exhibited the LOD of 0.3 ppm and showed the detection of a low amount of Ni^2+^ ions in water ranging from 0.1 to 1.0 ppm.

In optothermal applications, Hu and colleagues demonstrated a multi-layered bimetallic bactericidal nanoprobe comprising a core–shell–shell (Au–Ag–Au) structure for photothermal heating-mediated controlled release of Ag^+^ ions [133]. This bactericidal nanoprobe combined two features of photothermal sterilization based on the outer Au shell as well as the antibacterial effect of the inner Ag shell or Ag^+^ ions against surrounding bacteria. The outer shell can be melted even at low-power NIR laser irradiation (785 nm, 50 mW/cm^2^). The melting of the shell exposes the inner Ag shell, facilitating the release of antibacterial Ag^+^ ions. The bactericidal rate of 100% was observed in *E.coli* O157: H7 at 10 g/mL concentration of nanoprobes under 20-min irradiation. By exploiting the toxicity of Ag, the photothermal approach may alleviate the abuse of broad-spectrum antibiotics. In vivo biomedical application is another avenue that the photothermal method seems promising. A similar approach with Au–Ag core–shell nanospheres and NIR femtosecond laser pulse has been reported, wherein their superior photothermal-induced antibacterial activity was explored [134]. Positively charged Au–Ag nanosphere (19 nm in Au core; 3 nm in Ag shell) were attached to the negatively-charged bacterial surface via electrostatic interaction, forming large clusters on the surface of *S. aureus*. The NIR irradiation of Au–Ag nanospheres generated heat and ROS. As a result, Au–Ag nanospheres exhibited a strong antibacterial activity, as low as 7.5 pM in minimum inhibition concentration (MIC) against *S. aureus*. The result also showed a removal of up to 85% of a notoriously recalcitrant bacterial biofilm within 4 min under NIR irradiation.

Another example is shown by Kamimura and co-workers. The authors described surface-plasmon induced photocatalytic activity based on core@shell (Au–Ag) NPs [135]. Au@Ag NPs and Au–Ag bimetallic NPs were synthesized by multistep citric reduction and photo-reduction methods, respectively. Both types of metallic NPs exhibited strong absorption in the visible wavelength due to localized LSPR of Ag. They both could oxidize 2-propanol to acetone and CO_2_ under visible light irradiation (440–800 nm), but Au@Ag exhibited higher turnover rate than Au–Ag NPs. It was found that the improvement in chemical stability of Ag was attributable to the formation of a core@shell structure, which led to the efficient surface plasmon-induced photocatalytic activity.

Metallic nanospheres of Ag or Au has been utilized in optoelectronic light harvesting based on the plasmonic effect [136,137]. In plasmon-assisted solar energy conversion, metal nanostructures are used to scatter solar radiation and better able to couple radiation to semiconductor photovoltaic elements. The efficient extraction of light from LED exploits similar physics where the metal nanostructures play dual roles as a light scatterer and energy-transducing nanoantennas [8]. In this context, the solar cells serve to efficiently couple incident light to the AgNPs, from which optical energy propagates as surface plasmon (SP) polariton [138]. For example, the plasmonic effect triggered by metal NPs was used to enhance the yield of light absorption in solar cells [139,140]. As the photons of incident light were encountered by AgNPs, they caused electron vibration and scattering in the NPs, facilitating more efficient photon absorption. Rho and colleagues have reported dye-sensitized solar cells with AgNPs-decorated TiO_2_ nanotube arrays [141,142]. The energy conversion efficiency of the solar cells increased up to 32% by incorporating AgNPs into the TiO_2_ film. Another example is polymer optoelectronic devices with carbon dot-supported AgNPs (CD-AgNPs) which were described by Choi and co-workers [143]. The SPR effect of CD-AgNPs allowed additional light absorption and a significant amount of radiative emission in polymer solar cells as well as in polymer light-emitting diodes (LEDs).

## 5. Conclusions

AgNPs are emerging as a next-generation application in numerous subfields of nanomedicine, and potential benefits of using AgNPs as a prominent nanomaterial in biomedical and industrial sectors have been widely acknowledged. The comprehensive research regarding silver nanomaterials has been explored in this review to understand the synthesis methods and mechanisms, characterization of physicochemical properties, and possible toxicity and to discover more promising applications in oncology, personalized healthcare, and pharmacology. Among the various synthesis methods, biological green synthesis draws our attention as a promising alternative, due to its safety using natural agents and nontoxic chemicals. Diverse applications of AgNPs as plasmonic nanoantenna and biomedical and optoelectronic probes were also highlighted. Lastly, a better understanding of the cytotoxic mechanisms of AgNPs merits future research to broaden their nanomedical applications in diagnostics, therapeutics and pharmaceutics.
